# Evaluation of Senior Community Centers in Rural and Urban Pennsylvania

**DOI:** 10.1093/geroni/igaa057.116

**Published:** 2020-12-16

**Authors:** Janet Melnick, Heather Shanks-McElroy

**Affiliations:** 1 Pennsylvania State University, Dunmore, Pennsylvania, United States; 2 Keystone College, LaPlume, Pennsylvania, United States

## Abstract

This is a state-wide study of rural and urban Senior Community Care Centers in PA. The study focused on five key outcomes: To create an inventory of PA’s rural and urban SC locations, To analyze SC attendance and program participation, To analyze the challenges and opportunities that SCC’s face in providing services in rural and urban PA for a growing senior population. To identify innovative and successful models of senior community care centers within the state. To formulate policy recommendations for the state. Particular attention was placed on innovative programs who are attracting and serving the needs of Baby Boomers,

